# Quality of health care and patient satisfaction in liver disease: The development and preliminary results of the QUOTE-Liver questionnaire

**DOI:** 10.1186/1471-230X-8-25

**Published:** 2008-06-20

**Authors:** Jolie J Gutteling, Robert A de Man, Jan JV Busschbach, Anne-Sophie E Darlington

**Affiliations:** 1 Department of Medical Psychology and Psychotherapy, Erasmus MC, Rotterdam, The Netherlands; 2 Department of Hepatology and Gastroenterology, Erasmus MC, Rotterdam, The Netherlands

## Abstract

**Background:**

Consensus on how to adequately measure patient satisfaction with health care is limited, and has led to the development of many questionnaires with various methodological problems. The objective of this study was to develop a liver disease- and care-specific patient satisfaction instrument on the basis of previously tested methodology in patient satisfaction measurement, the so called QUOTE- series: Quality Of health care services Through the patients' Eyes. QUOTE methodology aims to standardise the measurement of satisfaction as the discrepancy between patients' needs, and the extent to which these needs are being met.

**Methods:**

As part of the QUOTE methodology routine, 11 Patients with chronic liver disease from the Erasmus MC (Rotterdam, the Netherlands) participated in focus-group meetings on patient satisfaction with the provided service at the outpatient hepatology clinic. Twenty-eight other patients were invited to rank the items generated during the focus-group meetings according to importance. With this information, the QUOTE-Liver was constructed. Face validity, construct validity, content validity, and reliability of the newly developed questionnaire were assessed in a test sample of 152 patients with chronic liver disease.

**Results:**

Two liver-disease specific, and the 18 items ranked as most important were included in the QUOTE-Liver. Face validity and content validity were acceptable: neither patients (n = 152) nor psychologists (n = 3) or a hepatologist suggested any extra items to be included. Construct validity was good: the overall score correlated significantly with the Visual Analogue Scale (VAS) measuring overall satisfaction (r = 0.69, p < 0.01). The reliability of the QUOTE-Liver was excellent (α = 0.90).

**Conclusion:**

The QUOTE-Liver is an easy to complete instrument based on standardized state-of-the-art satisfaction measurement methodology. Preliminary evidence for its validity and reliability was demonstrated. The QUOTE-liver covers those aspects of satisfaction that CLD patients consider to be important when visiting the outpatient department of hepatology. Even though further substantiating of the favourable psychometric findings is desirable, it seems to be a useful instrument that can be used to identify those aspects of care that need improvement in order to optimise the provision of health care for patients with chronic liver disease.

## Background

As liver disease is often chronic and progressive, frequent monitoring of medication and progression of the disease is necessary. Therefore, besides the quality of the medical therapy, good quality of care is important to these patients as they frequently interact with their physicians. Besides good medical therapy, good quality of care determines patient satisfaction. Patient satisfaction, in turn, has proven to be important in compliance with treatment, seeking medical advice and maintenance of a continuous relationship with a physician [1,2]. Also, patient satisfaction and quality of care are increasingly of interest to health insurance companies or health maintenance organisations that wish to negotiate prices when purchasing health care.

Although studies on quality of care/patient satisfaction are numerous, there has until recently been no consensus on how to measure this concept. Most researchers have unjustly dealt with patient satisfaction as an easy concept to measure [3]. This has led to several methodological problems in this field of research. First, the items of the questionnaires or inventories of patients' experiences, have been generated by health care professionals rather than patients, even though several studies have demonstrated that patients' and health care professionals' views about important aspects of satisfaction are often quite different [4-6]. Secondly, there has been a lack of disease- and care-specific items [7]. Finally, when asking patients to answer on a Visual Analogue Scale (VAS) the question "how satisfied are you", 90% tends to be satisfied in [7-9]. This may not be realistic, since a previous study has shown that many patients who reported being satisfied with the care they received, also indicated numerous problems with this same care [10]. Furthermore, this single item question does not give any indication of what needs to be changed when patients are not satisfied.

The lack of consensus on adequate measurement of patient satisfaction has led to a proliferation of questionnaires with inadequate measurement properties. A meta-analysis [3] in which 195 articles about patient satisfaction were screened, revealed that the instruments used to measure patient satisfaction were different in almost every study. Data on psychometric properties of the instruments, including their validity and reliability were scarce. A study on the use of patient satisfaction instruments by leading academic medical centres in the United States of America showed little standardization of the instruments currently being used at these centres, particularly for outpatient care [11].

The existence of so many different 'satisfaction' instruments and the lack of data on their reliability and validity does not only cast doubt on the soundness of the instruments, but also makes comparisons of studies impossible and conclusions drawn from studies implausible. For these reasons, the Netherlands Institute for Health Services Research (NIVEL) has developed a methodology protocol to develop a standardized series of questionnaires measuring *Qu*ality *o*f Care *T*hrough the Patient's *E*yes (QUOTE), based on market research theory [12], which asserts that consumer satisfaction should be measured by looking at the discrepancy between what consumers need/expect and what they actually receive [13]. QUOTE instruments are now available for a variety of diseases, such as HIV, IBD, and rheumatism. QUOTE instruments consist of two parts: the weight (importance) patients assign to different aspects of health care, second, patients' experiences with health care (performance). From the combined effect of importance and performance, the quality index can be obtained [14].

The aim of this study was to develop a liver disease- and care-specific version of the QUOTE, called the QUOTE-Liver, which measures quality of care/patient satisfaction in patients with chronic liver disease (CLD). The need for a liver disease- and care-specific instrument was grounded in the fact that CLD affects many people worldwide (560 million people are infected with the hepatitis B or C virus [15]), and alcohol-induced end-stage liver disease forms the second most important reason for liver transplantation in the United States [16]. CLD is a serious disease that is associated with significant physical and psychological symptoms such as impaired cognition, hepatic coma, fluid in the abdomen, abdominal pain, joint pain, fatigue, depression and anxiety [17-23]. The development of the QUOTE-Liver was undertaken in order to deal with all aforementioned methodological issues by asking patients rather than health care professionals to define important aspects of care, by including disease- and care-specific items, by focussing on the discrepancy between individual patients' needs and whether these needs have been met, and having been developed along the lines of a series of similar questionnaires which makes comparison between patient populations possible.

## Methods

In order to develop the QUOTE-Liver we followed the QUOTE protocol as described by the Netherlands Institute of Health Services Research (NIVEL, Utrecht, the Netherlands) [24].

### Study population

All patients suffering from chronic liver disease (CLD) visiting the outpatient department of Hepatology of the Erasmus Medical Centre in Rotterdam, which is a specialized centre for chronic liver disease in the Netherlands, during the first three months of 2004 were invited to participate in the development of the QUOTE-Liver by mail. Patients in a given period of time were made aware of the study by letter and invited to contact the researcher if they wanted additional information and if they were considering participating in the study. The phases of the study were done consecutively, each time approaching a new patient sample. Patients were included in each phase of the study on a first-come first-serve basis. Consequently, no information is available on those patients who did not want to take part in the study. Patients who were willing to participate were contacted by the researcher, who provided them with verbal and additional written information. All participants completed an informed consent form. No incentives for participating in the study were given. Travel expenses of the patients participating in the focus group discussions were covered. The study protocol was in accordance with the ethical guidelines of the modified 1975 Declaration of Helsinki. Since patients were invited to participate in one part of the study only, and since the study did not include invasive questions, ethical approval was not necessary under Dutch regulations.

### Part one: Focus groups

Focus groups were first mentioned as a market research technique in the 1920's [25,26]. They are an efficient means to obtain data on opinions and attitudes. Focus groups are qualitative interviews with a small number of people. Unlike one-on-one interviews, focus groups generate information through group discussion, which besides information about what people think, gives insight in why people think the way they do. For good results, just a few focus groups are sufficient, as data become saturated and little new information emerges after the first few groups [27].

The purpose of the focus groups in this study was for patients to generate a list of relevant care aspects at the outpatient department of Hepatology of the Erasmus MC. Three focus group meetings were organized at the Erasmus MC (Rotterdam, the Netherlands), in which 11 patients participated (table 1). During the focus group meetings, patients were asked to name all aspects of their visit to the outpatient department of Hepatology that were important to them. Meetings lasted from 70 to 90 minutes. Two researchers, one psychologist and one experienced psychotherapist who could resolve any arising emotional issues, conducted the focus groups.

**Table 1:** Demographic and clinical characteristics of the patient population included in the development of the questionnaire.

	**Focus Groups**	**Item Ordering**	**Validation study**
Total number of patients (n)	11	28	152

Male (n, %)	6 (55)	11 (39)	81 (53)
Age (mean, range)	48.3 (18–75)	50.7 (21–74)	46.7 (19–75)
Liver disease (n,%)			
Post-transplantation	5 (45)	5 (18)	24 (16)
Viral Hepatitis	4 (37)	10 (36)	72 (47)
Cholestatic Liver Disease	2 (18)	3 (10)	40 (26)
Other	0 (0)	10 (36)	16 (10)

### Part two: Item Ranking

The patients ranked the items to create an order of importance in the aspects named in the focus group meetings [28]. For this ranking exercise, which took place at the outpatient department of Hepatology of the Erasmus MC, all aspects mentioned during the focus group meetings were written on separate cards, which patients were asked to divide over five piles, ranging from "most important (1)" to "least important (5)". The piles had to be of nearly equal size in order to force patients to make a choice. To make sure that no aspects had been missed during the focus group meetings, the patients were asked at this stage whether they thought any important items were missing. The item-ranking task was carried out by one researcher.

### Part three: Selection of items

The QUOTE protocol has no strict guidelines regarding the number of items to be included. Because we wanted an easy to administer questionnaire, we (the authors) opted for a 20-item questionnaire, which is in the lowest range of item numbers of the already existing QUOTE instruments like the QUOTE-IBD (23 items), QUOTE-HIV (27 items), QUOTE-Occupational Therapy short version (23 or 12 items). Since we were interested in the items that were most important to patients, we decided to follow the preferences of patients closely, rather than to chose items belonging to a priori determined aspects of care such as accessibility, waiting room area, etc. Because QUOTE questionnaires are disease- and care-specific, disease- and care-specific items were also included.

#### Scoring and interpretation of scores

All QUOTE questionnaires, and thus also the final QUOTE-Liver, consist of two parts. The questioning style is deliberately repetitious as it has been reported that this ensures patients' understanding. In part one, importance of the 20 items is measured. The fact that ordinary Likert scales tend to be highly skewed towards the 'important' dimension was solved by providing 4-point response options (0 = 'not important at all', 3 = 'slightly important', 6 = 'important', 10 = 'very important'), which proved to be a workable solution [14]. In part two, performance on those same 20 items is measured. Patients are asked what actually happened during the consultation. They can rate items on a four-point scale ranging: 'no' (score = 1), 'not really' (0.67), 'mostly yes' (0.33), and 'yes' (0). The QUOTE protocol defines the aspects of care that need improvement when more than 10% of patients are dissatisfied, reflected by a total score of <9.0 (range is 0 – 10), computed by the formula: 10 – importance × performance [29]. For the QUOTE-Liver we have followed this QUOTE family approach to item scaling.

### Part four: Validation study

#### Psychometric methods

*Principal component analysis *was run to explore factors within the questionnaire.

*Reliability *of the QUOTE-Liver was measured by computing the internal consistency of the items and the item-total correlations using the reliability analysis in SPSS 11.0. *Face validity*, i.e the extent to which experts judge the instrument to measure the intended concept, was determined by presenting it to 152 patients with chronic liver disease, three psychologists and a hepatologist. To guarantee correct measurement of the concept (quality of care from patients' perspective), construct and content validity were measured. *Construct validity*, i.e. the degree to which an instrument measures the theoretical construct it is intended to measure, was measured by computing Pearson correlations between a Visual Analogue Scale (VAS), and the total quality impact score of the QUOTE-Liver. The VAS consisted of a horizontal line on which patients had to indicate by means of a cross, to what extent they agreed (no, definitely not – yes, definitely) with the following question: 'Would you recommend a consultation at this outpatient department to your best friend if he/she was in the same circumstances?" Scores were calculated as percentages of the scale, with 0 indicating total disagreement, and 100 indicating perfect agreement. To assess *content validity*, patients were asked to indicate which other items should be included in the QUOTE-Liver. A researcher who was present while patients completed the questionnaire explored the *feasibility *by looking at the time it took for patients to complete the questionnaire, by reporting patients who failed to complete the questionnaire, and by noting patients' questions regarding the instructions and/or items. In addition, patients were asked whether they had understood the questions, if they thought completing the questionnaire was easy or difficult, and what they thought of the completion time.

## Results

### Patients in the study

11 patients with CLD participated in the focus group meetings, and 28 patients participated in the item-ranking task. 152 patients completed the QUOTE-Liver for validation purposes. The demographic and clinical characteristics of these patients are shown in Table 1.

### Part one: Focus groups

The focus group meetings generated 121 partly overlapping aspects that patients found important when visiting the department of Hepatology. These were converted into 70 distinct items. Most items (30) concerned competence and social skills of the physician. 16 items concerned the waiting room area, nine items referred to assisting personnel and making appointments, eight items concerned availability of information, two were about venipuncture and five concerned accessibility of the hospital.

### Part two: Item ranking

The mean importance scores of the 70 items ranged from 4.65 for competence of the physician to 1.38 for presence of a cloakroom at the outpatient department. Two disease- and care-specific items were mentioned: "the venipuncture nurses are skilled" and "I don't have to wait long for venipuncture". No items were mentioned when patients were asked if any important items were missing.

### Part three: Selection of items

Two disease- and care-specific items were included in the QUOTE-Liver: 'waiting time for venipuncture' and 'skills of the venipuncture nurses'. Besides these two items, the 18 most important items following from the item-ranking task were included. The importance scores did not show a clear difference between important and less important items. Rather, scores for importance decreased gradually (score of item 18 = 3.88, score of item 19 = 3.81). Consequently, the decision to include 20 items in the QUOTE-Liver was made to coincide with the number of items of other QUOTE-instruments. Table 2 shows all items included in the QUOTE-Liver. The scores presented in this table were derived from the validation study conducted with 152 patients. In that study, the average importance and performance scores were computed using the Likert scales, resulting in a range varying from 0 to 10 for the importance scores and a range of 0 to 1 for the performance scores. There were no missing data.

**Table 2:** Items included in the QUOTE-Liver and scores of 152 Dutch patients with chronic liver disease.

		Importance Score	Performance Score	Quality Impact Score
*How important is it to you that the doctor that you visit today*...				
1	Is knowledgeable	9.46	0.04	9.60
2	Takes time to discuss emotional issues	6.58	0.15	9.21
3	Takes you seriously	9.00	0.04	9.66
4	Makes you feel safe	8.09	0.07	9.50
5	Believes what you say	8.50	0.05	9.57
6	Takes enough time for you	8.05	0.07	9.45
7	Is friendly	7.53	0.03	9.81
8	Is open	8.47	0.05	9.58
9	Listens to you	8.58	0.05	9.59
10	Answers all of your questions	8.67	0.06	9.51
11	Gives you enough information about your disease/treatment	9.01	0.08	9.31
12	Gives you a say in your treatment	7.76	0.10	9.24
13	Answers your questions clearly	8.56	0.07	9.45
14	Gives you medical/technical information about your disease when you ask for it	8.41	0.05	9.62
15	Gives enough explanation about your medication and possible side effects	8.24	0.05	9.56
16	Refers you well when you present with complaints that are not liver disease-related	8.03	0.04	9.61
17	Takes action quickly	8.96	0.04	9.62
*How important is it for you that*...				
18	You can tell your doctor what's on your mind	7.15	0.05	9.72
19	The venipuncture nurses are skilled	7.78	0.05	9.54
20	The waiting time for venipuncture is short	5.47	0.12	9.12

The QUOTE-Liver consists of the two disease- and care-specific items (19 and 20) and the 18 most important items for quality of care as measured in a population of chronic liver patients. The questioning style is deliberately repetitious, as it has been reported that this ensures patients' understanding. Importance scores can range from 0–10, performance scores can range from 0–1. A 'quality impact score' of <9.0 indicates that more than the usual 10% of the patients are dissatisfied with the particular aspect of care.

### Part four: Validation study

#### Data analysis

Principal component analysis yielded four factors. 17 items concerning interaction/contact with the physician loaded high on factor one. One disease- and care-specific item ("How important is it for you that the waiting time for venipuncture is short") loaded high on factor two. The other disease-and care-specific item ("How important is it for you that the venipuncture nurses are skilled" loaded high on factor three. One item ("How important is it to you that the doctor that you visit today is knowledgeable") loaded high on factor four.

The internal consistency of the overall QUOTE-Liver was excellent (α = 0.90). The item-total correlations of the 17 items concerning interaction with the physician ranged from 0.40 to 0.71 (alpha if item deleted = 0.89 – 0.90). The item-total correlation of the two disease- and care-specific items and of the first item concerning the physician's knowledge were 0.08, 0.37 and 0.39 respectively (alpha if item deleted = 0.90–0.91). Face validity was excellent: all patients (n = 152) in the validation study, and three psychologists and a hepatologist agreed that the items of the QUOTE-Liver adequately reflected the most important aspects of care for CLD patients. Construct validity, as measured by the correlation between the VAS measuring overall satisfaction and the total score on the QUOTE Liver was substantial (r = 0.69; p < 0.01). Content validity was also good: none of the 152 patients in the validation study suggested new items to be included. Feasibility was established as all patients in the validation study completed the QUOTE-Liver quickly (average time of 1.5 minutes), and understood the items.

## Discussion

In the present study, we have developed an easy to complete, self-administered liver disease- and care-specific questionnaire that measures quality of care through the patient's eyes (QUOTE-Liver). Preliminary evidence for its validity and reliability was demonstrated. The QUOTE-Liver was developed using a protocol that has recently been applied to develop a series of disease- and care-specific patient satisfaction instruments (QUOTES) [14]. The QUOTE-Liver consists of two liver disease- and care-specific items, and the 18 most important items out of 70 defined by 28 patients with chronic liver disease. Seventeen items concerned interaction/contact with the physician. Three other items did not correlate with the 17 items on interaction/contact with the physician. These items were added to the questionnaire since they had clear theoretical and clinical relevance and added to the disease-specific nature of the QUOTE-Liver questionnaire. Future research would do well to further look into these three items and further elaborate on them.

It is notable that nearly all items included in the QUOTE-Liver concerned aspects related to the physician. Apparently, other aspects of care such as accessibility, cooperation with other health care workers, and accommodation are of lesser importance to patients with chronic liver disease. This may explain the fact that no dissatisfaction, as shown by a score below 9.0, was established. Indeed, other QUOTE instruments found dissatisfaction on items pertaining to accessibility (by telephone) [8,29-32], doctors' and nurses' psychosocial approach [29,30,32], information [8,29-32], cooperation with other health care workers [30-32], privacy [8,32], and patient authority [8,30], rather than medical competence, contact, and communication, with the exception of 'information'. Exclusion of these multiple aspects in the development of the QUOTE-Liver was chosen for deliberately since these were of lesser importance to patients with chronic liver disease.

The high satisfaction scores obtained by the QUOTE-Liver in the present study may have been a result of the high standards of care at the specialized liver center of the Erasmus MC. However, these high scores cast doubt on its ability to detect change over time in the same group receiving an intervention, or changes between groups receiving different interventions. As the instrument could potentially be important for use in intervention studies, further testing of the QUOTE-Liver in its current form, preferably in an experimental setting where "bad" care is delivered purposefully, is needed in order to draw firmer conclusions on its sensitivity. Another way to assess the sensitivity of the QUOTE-Liver may be to administer it in two different settings, a specialized setting and a less specialized setting. A before and after study, where some aspect of care has been changed, is also a possibility that should be explored.

The method of patient selection used in this study may have caused bias. Even though the most important forms of chronic liver disease (HBV, HCV and cholestatic liver disease) were represented in the patient sample used in the present study, and even though the average age was representative of the overall population of CLD patients [17], no information was available of patients who did not want to participate. Future studies should register characteristics of non-participants.

Future studies should also assess the reproducibility of the QUOTE-Liver in terms of test-retest reliability. In addition, other ways should be explored to assess the construct validity of the QUOTE-Liver. A VAS measuring satisfaction was chosen since no adequate alternative was available at the time of the study. Measuring satisfaction by means of a VAS is relatively crude and possibly influences the subsequent construct validity for which it was used. Future studies could use the generic Dutch version of the QUOTE, which is now available, to assess construct validity.

A possible limitation of QUOTE instruments in general that has so far never been mentioned, is that some patients may be reluctant to say "no" (e.g. no, my physician did not discuss emotional problems) as it may reflect badly on the physician. Indeed, some patients in our study expressed a need for an answering category 'not relevant' for certain items of part two of the QUOTE-Liver. This should certainly be considered, as it will probably increase patient participation without compromising the scoring of the instrument.

## Conclusion

In conclusion, the QUOTE-Liver is an easy to complete instrument to assess liver patients' satisfaction with health care at an outpatient department, with evidence of good validity and reliability. The high satisfaction scores that the QUOTE-Liver produced in our validation study may be a result of the items included in the questionnaire, which mostly address physician competence and social behavior. As long as physicians' treatment and communication are good, high scores are to be expected. Further studies are needed to test the responsiveness and generalizability of the QUOTE-Liver to other linguistic and cultural settings.

## Competing interests

The authors declare that they have no competing interests.

## Authors' contributions

JJG: Has been involved in design of the study, data collection, statistical analyses and drafting of the manuscript. A–SED: has made substantial contributions to interpretation of data and has been involved in drafting the manuscript. JJVB: has been involved in revising the intellectual content. RAdM: has been involved in data collection, and revision of the intellectual content. All authors have read and approved the final manuscript.

## Pre-publication history

The pre-publication history for this paper can be accessed here:


